# 

**Published:** 1915-05-08

**Authors:** 


RATES OF SUBSCRIPTION: Twelve months, 6/6; Six months, 4/-; Three months, 2/-, post free to any address in the United Kingdom. Abroad, Twelve months, 8/8; Six months, 5/-; Three months, 2/6, post free.—Address The Subscription Dept., "THE HOSPITAL," The Hospital Building, 28/29 Southampton Street, Strand, London, W.C.

